# Tracheostomy Outcomes on Trauma Patients

**DOI:** 10.1002/oto2.48

**Published:** 2023-04-13

**Authors:** Edgar Del Toro‐Diez, Camila S. Ríos De Choudens, Shayanne A. Lajud, Jeamarie Pascual‐Marrero, Adriana Baez‐Bermejo

**Affiliations:** ^1^ Department of Otolaryngology–Head and Neck Surgery, School of Medicine University of Puerto Rico San Juan Puerto Rico USA; ^2^ Department of Otolaryngology–Head and Neck Surgery University of Toronto Toronto Canada; ^3^ Department of Pharmacology, School of Medicine University of Puerto Rico San Juan Puerto Rico USA

**Keywords:** decannulation, open surgical tracheostomy, percutaneous dilatational tracheostomy, Puerto Rico Medical Center, tracheal stenosis, tracheostomy complications, tracheostomy tube size

## Abstract

**Objective:**

Tracheostomies are performed in trauma patients for multiple purposes. Approaches to the procedure are usually directed by individual expertise and local preferences. Though generally safe, a tracheostomy can cause serious complications. This study aims to identify complications associated with tracheostomies performed at the level I Trauma Center of the Puerto Rico Medical Center (PRMC) to have an advanced foundation to develop and implement guidelines to improve patient outcomes.

**Study Design:**

A retrospective cross‐sectional study.

**Setting:**

Level I Trauma Center of the PRMC.

**Methods:**

Medical charts of 113 trauma adult patients that underwent tracheostomy at the PRMC from 2018 to 2020 were reviewed. Data collected included patient demographics, surgical approach, initial tracheostomy tube size (ITTS), intubation period, and flexible laryngoscopic findings. Complications occurring during and after tracheostomy were documented. The unadjusted relationship of the independent variables and outcome measures was assessed using *χ* ^2^ and Fisher's test for categorical variables and the Wilcoxon‐Mann‐Whitney rank‐sum test for continuous ones.

**Results:**

Abnormal airway findings detected on flexible laryngoscopic examination were reported in 30 patients in the open tracheostomy (OT) group and 43 patients in the percutaneous tracheostomy group (*p* = 0.007). Peristomal granulation tissue was reported in 10 patients with an ITTS 8, while in only 1 patient with an ITTS 6 (*p* = 0.026).

**Conclusion:**

This study showed several key findings in our cohort. The OT surgical approach was associated with fewer long‐term complications when compared to the percutaneous approach. Also, a statistically significant difference in peristomal granulation tissue findings was found between the ITTS, ITTS‐6 and ITTS‐8, the smaller size being associated with fewer abnormal findings.

Tracheostomies transformed the care of trauma patients by facilitating ventilation in patients with airway compromise, easing the weaning process from the ventilator, and decreasing ventilator or orotracheal tube‐related complications, among other reasons.

General indications for tracheostomy placement include acute respiratory failure, upper airway obstruction, failure to wean from mechanical ventilation, and abundant secretions.^1^, ^2^, ^3^ The average number of tracheostomies performed in the United States is estimated to be approximately 100,000 annually.^4^ However, a tracheostomy is not without complications, including surgical, medical device‐related, or tracheostomy care complications. All of these may present in the acute or chronic setting and can be life‐threatening.^5^, ^6^ Tracheostomies have also been associated with longer hospital stays, decreased quality of life, and social isolation.^4^, ^7^, ^8^ Importantly, they have a direct and indirect economic impact on the patient and the health services.^9^, ^10^ Approximately, 20% of patients with a tracheostomy are discharged with the tracheostomy in situ.^11^

Although a tracheostomy is one of the most frequently performed lifesaving surgical procedures on critically ill patients, there is no consensus among different specialties regarding a universally accepted approach. Recently, similar complication outcomes have been published between open tracheostomy (OT) and percutaneous tracheostomy (PT) approaches.^12^ However, limited research has been published regarding outcomes associated with initial tracheostomy tube size (ITTS) use. Research shows that Hispanic adults are at an increased risk of death from a tracheostomy complication compared with the general US population.^5^ The reasons leading to the increased mortality are yet to be established. Data in Puerto Rico on the incidence and prevalence of the procedure and its associated outcomes and complications are limited. The Puerto Rico Medical Center (PRMC) is the main quaternary referral academic health center. Every year, between 200 and 300 patients (adults and children) with tracheostomies receive follow‐up treatment at the Otolaryngology–Head and Neck Surgery clinics. This study aims to identify and compare the primary practices used to perform tracheostomy in the PRMC level 1 trauma center through a retrospective chart review study. Our study also aims to evaluate outcomes associated with the identified techniques. We hypothesized that each specialty has a favored method of performing a tracheostomy. We also hypothesized that there would be a difference in complication rate among ITTS of different sizes.

## Methods

We conducted a cross‐sectional historical study by reviewing the medical records of 146 trauma patients that underwent tracheostomy between 2018 and 2020 at the level I Trauma Center at the PRMC. Included in the study were patients 20 years and older (legally adults in Puerto Rico) based on the International Classification of Disease codes Z93.0, J95.00, J95.01, J95.02, J95.03, J95.04, and J95.0. Excluded from the study were patients 19 years old and younger, those not evaluated for decannulation by our service, and those whose records were incomplete. Patients with cricothyrotomy were not included due to the small number of patients that underwent this procedure. The study protocol was approved by the MSC‐Institutional Review Board (Protocol B1660121).

Tracheostomies were performed by trauma surgeons, and ultimately, all patients were consulted with the Otolaryngology service to evaluate candidacy for decannulation. Patients were evaluated per our protocol for decannulation, which consists of medical history and a physical examination that includes a flexible laryngoscopic evaluation with and without the tracheostomy tube to evaluate the airway down to the bronchi for potential abnormal airway findings that impede decannulation. Data were extracted from the hospital's electronic medical records. It included patient demographics, intubation period, surgery date and surgical approach, ITTS, capping days, and flexible endoscopy findings such as aspiration, tracheal or subglottic stenosis, and granulation tissue, among others. This study's abnormal airway findings refer to any deviation from normal pharyngeal and laryngotracheal anatomy. These include tracheal or subglottic stenosis, tracheal granulomas, fractured and exposed cartilage, and vocal fold palsies.

Descriptive statistics were used to describe the patient's characteristics in frequencies and percentages. Independent variables were the surgical approach and ITTS. Outcome measures included decannulation rates and abnormal airway findings (airway lesions and stenosis). The unadjusted relationship of the variables in these groups was assessed through *χ* ^2^ and Fisher's test for categorical variables and the 2‐sample Wilcoxon‐Mann‐Whitney rank‐sum test for continuous ones. A *p* value of 0.05 was considered statistically significant. Statistical analysis was performed using Stata 14.2 (StataCorp).

## Results

### Demographics

Baseline demographics are presented in Table 1. Of the 146 patients who met the inclusion criteria, 113 (77.4%) were included, and 33 were excluded due to missing data. Their mean age was 46 ± 18.3 years. The majority (92, 81.4%) were male, and 21 (18.6%) were female. The subjects' mean body mass index (BMI) was 28.99 ± 8.2, and 28.2% were obese. Almost half of the patients had toxic habits; 48.5% were alcohol users, 35.8% were smokers, and 21.21% were drug users. The most common comorbid conditions, reported in half (53.9%) of the cohort, were asthma, type 2 diabetes mellitus, and high blood pressure.

**Table 1:** Demographics of Tracheostomized Patients

	n = 113	Percentage (%)
Age, years (median, SD)	46.21 (18.3)	
Gender		
Female	21	18.6
Male	92	81.4
BMI (median, SD)	29.00 (8.2)	
BMI > 30	24	28.2
Comorbidities	56	53.9
Alcohol abuse	33	48.5
Smoking	24	35.8
Drug abuse	14	21.2

Abbreviations: BMI, body mass index; SD, standard deviation.

### Clinical Characteristics

The clinical characteristics of the tracheostomy cohort are shown in Table 2. The most common indication for performing tracheostomy was the need for prolonged mechanical ventilation (106 patients, 93.81%). Other reasons for tracheostomy included respiratory failure, airway compromise, and avoidance of complications. The mean intubation period was 20 (±11.0) days. The mean tracheostomy duration was 70 (±13.27) days (not included in Table 2). The 2 surgical approaches used were comparable in numbers: the open tracheostomy (OT) was used in 57 (50.4%) patients, and the percutaneous dilatational tracheostomy (PDT or PT) was used in 56 (49.6%) patients. Forty‐six (40.7%) patients received a cuffed adult ITT size 6.0 (ITTS‐6), while the remaining patients (58.4%) received a cuffed adult size 8.0 tracheostomy tube (ITTS‐8). At the time of the Otolaryngology service evaluation for decannulation, 89 patients had been transitioned to an adult cuffless tracheostomy tube size 6.0 (92.7%), 2 patients had an adult cuffless tracheostomy tube size 4.0 (2.1%), and 22 patients had an adult cuffless tracheostomy tube size 8.0 (5.2%).

**Table 2:** Clinical Characteristics of Patients Undergoing Tracheostomy

Reason for tracheostomy	Total (n = 113)	Percentage (%)
Airway compromise	2	1.8
Avoidance of complication	1	0.9
Prolonged mechanical ventilation	106	93.8
Respiratory failure	4	3.5
Surgical approach		
Open tracheostomy	57	50.4
Percutaneous tracheostomy	56	49.6
Initial tracheostomy tube size (ITTS) placed during surgery		
ITTS 6	46	40.7
ITTS 8	66	58.4
Tracheostomy tube size (TTS) upon otolaryngology evaluation		
TTS 4	2	2.1
TTS 6	89	92.7
TTS 8	5	5.2
Cuffed tracheostomy at the time of otolaryngology evaluation	6	5.9
Cuffless tracheostomy at the time of otolaryngology evaluation	96	94.1

Table 3 shows the complications detected according to the surgical approach. The complications identified were similar for both surgical approaches. There was no difference in the frequency of dysphonia between each group. However, there was a statistically significant difference (*p* = 0.007) in total abnormal findings detected in the PDT group (n = 43) as compared to the OT group (n = 30). In the PDT group, a higher number of patients had lesions or abrasions, vocal cord dysfunction, granuloma, nonpatent airway, aspiration, airway narrowing, and lesions/masses on retroflex and dysphagia. When subgroup analysis was performed, these differences were not statistically significant.

**Table 3:** Abnormal Findings According to Surgical Technique

Finding	OT (N, %)*	PDT (N, %)*	*p* value
Dysphagia	1, 25.0	3, 75.0	0.618
Dysphonia	14, 50.0	14, 50.0	1.00
Peristomal granulation tissue	7, 63.6	4, 36.6	0.527
Abnormal FL or tracheoscopy findings	30, 41.1	43, 58.9	**0.007**
FL granuloma, abrasion, or lesion	14, 43.8	18, 56.2	0.339
Vocal cord dysfunction	19, 45.2	23, 54.8	0.354
Nonpatent airway	3, 42.9	4, 57.1	0.714
Aspiration	4, 40.0	6, 60.0	0.482
Airway narrowing	1, 25.0	3, 75.0	0.363
Lesions or masses on retroflex	13, 44.8	16, 55.2	0.388

*Note*: Bold value indicates statistically significance.

Abbreviations: FL, flexible laryngoscopy; OT, open tracheostomy; PDT, percutaneous dilatational tracheostomy.

*Data are presented as the sample size (n) and percentages.

Complications detected according to the tracheostomy tube size are listed in Table 4. Abnormal airway findings were more common in the ITTS‐8 group (n = 47) than those with ITTS‐6 (n = 20). Peristomal granulation tissue was detected in 10 patients with ITTS‐8 and 1 with ITTS‐6, showing a statistically significant difference (*p* = 0.026).

**Table 4:** Abnormal Findings According to Tracheostomy Tube Size

Characteristics	ITTS‐6 (N, %)*	ITTS‐8 (N, %)*	*p* value
Dysphagia	1, 25.0	3, 75.0	1
Dysphonia	11, 39.3	17, 60.7	0.821
Peristomal granulation tissue	1, 10.9	10, 90.1	**0.026**
Abnormal FL or tracheoscopy findings	25, 34.7	47, 65.3	0.067
FL granuloma, abrasion, or lesion	12, 37.5	20, 62.5	0.592
Vocal cord dysfunction	13, 31.7	28, 68.3	0.111
Nonpatent airway	1, 14.3	6, 85.7	0.238
Aspiration	3, 30.0	7, 70.0	0.488
Airway narrowing	1, 25.0	3, 75.0	0.641
Lesions or masses on retroflex	11, 37.9	18, 62.1	0.826

*Note*: Bold value indicates statistically significance.

Abbreviations: FL, flexible laryngoscopy; ITTS, initial tracheostomy tube size.

*Data are presented as the sample size (n) and percentages.

## Discussion

Our study showed that OT and PDT surgical approaches are used comparably in our clinical setting. Interestingly, our results show a statistically significant association between PDT and total abnormal airway findings detected on flexible laryngoscopy (Table 3). Such findings included tracheal or subglottic stenosis, tracheal granulomas, fractured and exposed cartilage, and vocal fold palsies. When subgroup analysis was performed for individual airway abnormalities, none yielded a statistically significant value. There is limited literature comparing abnormal sequela of OT versus PDT surgical approaches. Johnson‐Obaseki et al, in a meta‐analysis, compared both surgical approaches and reported no difference in mortality or hemorrhage rates.^12^ However, their evaluation of infection rates and operative time revealed a statistically significant difference in favor of PDT. Our study did not address these complications, though it warrants analysis. A recent review of complications of PDT and gastrostomy performed at the bedside in an intensive care unit highlights some complications, including posttracheostomy tracheal stenosis, tracheomalacia, and tracheoinnominate and tracheoesophageal fistulas.^13^ However, a prospective study comparing PDT and OT concluded that PDT is safe in the early term but has higher rates of long‐term complications.^14^ Norwood et al described the complications associated with PDT in a large cohort (n = 900 patients) and found that PDT is associated with dysphonia, tracheal stenosis, airway granulomas, and tracheomalacia.^15^ Although in a small cohort, our study confirms Norwood's results.

Regarding the tube size, complications identified were more frequent in the ITTS‐8 group (Table 4). The ITTS‐8 was statistically significantly associated with an increased prevalence of peristomal granulation tissue. This abnormality is most likely due to the traumatic insertion of a high‐caliber tube that might lead to cartilage exposure and irritation of the stoma. There is limited data in the literature concerning the ITT size and its complications. A study performed to determine the relationship between BMI to tracheal airway dimensions showed that as the BMI increased tracheal width appears to decrease.^16^ The authors suggested avoiding the tendency to use a larger tube to secure the airway of an obese patient due to an erroneous assumption associating obesity and trachea size. Further studies to validate these findings are warranted. Also, Li et al described an association between tracheal stenosis and PDT and an ITTS greater than 6.0.^17^ Recent literature on quality improvement highlights the importance of an interdisciplinary approach to tracheostomy care to decrease complication rates and length of stay, among many additional benefits.^18^, ^19^

### Limitations

This research is subject to several limitations. The study is limited by its scope as it is focused on an academic medical center, and results may not be generalizable to other contexts. The sample size is small since this is a retrospective analysis of tracheostomies performed at 1 site, PRMC, for 3 years. The sample size was determined by the population and the number of trauma incidents and likely influenced efforts to achieve statistically significant results.

## Conclusion

We identified some potential quality issues when comparing PDT versus OT that should be studied further. Our study demonstrated that in our cohort, a higher diameter tube causes peristomal granulation tissue and that abnormal airway findings were identified more commonly in patients who underwent PDT, which impacted the decision to decannulate patients. An indwelling tracheostomy due to its complications may lead to prolonged hospital length of stay, as well as affect the quality of life of the patients and caregivers, and increase hospitalization costs. Recruitment of patients for a research project to establish a tracheostomy registry is ongoing which will help us in achieving statistically significant data to address these issues and improve patient care in our setting.

## Author Contributions

**Edgar Del Toro‐Diez**, conducted the research, analyzed the data, and wrote the manuscript; **Camila S. Rios De Choudens**, designed and conducted the research, analyzed the data and wrote the manuscript; **Shayanne A. Lajud**, designed the research and provided feedback during manuscript development; **Jeamarie Pascual‐Marrero**, provided feedback during study design and manuscript development; **Adriana Baez‐Bermejo**, designed the research, analyzed the data, participated in manuscript elaboration, and approved the final document.

## Disclosures

### Competing interests

The authors have no conflicts of interest to declare.

### Funding source

Department of Otolaryngology–Head and Neck Surgery, School of Medicine, University of Puerto Rico.
